# Long-term success of percutaneously placed peritoneal dialysis catheters

**DOI:** 10.1080/0886022X.2026.2681247

**Published:** 2026-06-09

**Authors:** Alexis Lorio, Mahnoor Liaqat, George L. Parra, Claire Juhas, Manoj Bhattarai, Shweta Bansal

**Affiliations:** a Department of Medicine, Duke University School of Medicine, Durham, NC, USA; b Department of Medicine, Dell Medical School, University of Texas at Austin, Austin, TX, USA; c Graduate School of Biomedical Sciences, University of Texas Health San Antonio, San Antonio, TX, USA; d Department of Surgery, University of Oklahoma—Tulsa, Tulsa, OK, USA; e Department of Nephrology, University of Texas Health San Antonio, San Antonio, TX, USA

**Keywords:** Catheter-related events, long-term survival, peritoneal dialysis catheter, percutaneous insertion, surgical insertion

## Abstract

Percutaneous peritoneal dialysis (PD) catheters demonstrate short-term outcomes comparable to surgically placed catheters but remain underutilized because of concerns regarding long-term durability and limited follow-up data. We retrospectively reviewed 117 PD catheters in 90 patients who initiated PD between 2014 and 2020 at a freestanding home dialysis unit, with follow-up through December 2022. Eighty-three catheters were placed percutaneously by interventionalists and 34 laparoscopically for suspected hernias, prior abdominal surgery, or refractory peritonitis. The primary outcome was catheter survival, defined as time from PD training initiation to PD discontinuation. Secondary outcomes included catheter-related adverse events not requiring removal. The mean age was 46.5 ± 14 years; 38% were female, 76% Hispanic, mean BMI was 30 ± 7 kg/m^2^, and diabetes accounted for 63% of kidney failure cases. Baseline characteristics were similar between groups except for lower BMI in the percutaneous cohort. Median catheter longevity was 20.3 months (IQR 6.8–36.0) for percutaneous placement and 17.0 months (IQR 7.2–30.8) for surgical placement. PD-related infections were the leading cause of catheter removal in both groups, while the rates of major and minor mechanical complications were comparable. To our knowledge, this is among the largest contemporary real-world cohorts evaluating long-term outcomes of percutaneous PD catheters, with follow-up extending beyond 5 years, and it addresses an important evidence gap. Percutaneous PD catheters demonstrated long-term survival and complication rates comparable to those of surgically placed catheters. These findings support broader adoption of percutaneous catheter placement to facilitate timely PD initiation and patient-centered modality selection.

## Introduction

For patients with end-stage kidney disease (ESKD), home dialysis, particularly peritoneal dialysis (PD), has increasingly been advocated as a treatment modality owing to several clinical, quality of life, and cost benefits [1–4]. High-quality and functional dialysis access is paramount for successful initiation and maintenance of PD. PD catheter-related problems remain a major cause for the transfer of patients on PD to hemodialysis [5]. PD catheters can be inserted surgically (open and laparoscopic) by surgeons or percutaneously (under radiological or sonographic guidance) by interventional radiologists (IR) or nephrologists (IN) [6]. However, the advantages of one technique over the other continue to be debated. In the contemporary era, the peritoneoscopic method is almost obsolete. Percutaneous catheters offer the advantage of quick placement without much prior preparation or general anesthesia. On the other hand, surgical catheters offer the opportunity for direct visualization of the abdomen and potential lysis of adhesions and hernia repair; however, these are more invasive and require general anesthesia requiring cardiac clearance, imparting a higher risk to an already disadvantaged population.

Catheter-related problems include peri-catheter leaks, infections, migration, and dysfunction, which may require catheter removal [6]. Prior retrospective studies comparing surgical and percutaneous techniques have demonstrated variable complication rates and catheter survival, but overall outcomes have generally been considered comparable [7,8]. Despite these findings, percutaneously placed catheters remain an underutilized resource [9,10]. Importantly, most existing studies are limited by short follow-up durations, typically ranging from 6 to 12 months, leaving uncertainty regarding the long-term durability and safety of percutaneous catheter placement. At our center, catheters are placed percutaneously, with surgical (laparoscopic) placement reserved for patients with concerns of hernia or adhesions due to prior extensive abdominal surgery or refractory peritonitis. This practice provides a unique opportunity to evaluate long-term outcomes associated with percutaneous catheter placement in a real-world setting. We hypothesize that percutaneous PD catheter placement is associated with durable long-term catheter survival and acceptable complication rates over extended follow-up.

## Methods

### Study design and sample

We conducted a retrospective analysis of a patient population who initiated PD between March 01, 2014 and December 31, 2020 at a free-standing adult home dialysis facility at Texas Diabetes Institute, University Health, San Antonio, a large academic and safety net hospital system. The final follow-up date was December 31, 2022. This time frame allowed accessible patient charts in the electronic medical records and at least two years of follow-up data. Medical records were accessed in 2023. In the center’s practice, catheters are placed percutaneously either by IR or IN, unless there are concerns of hernia or adhesions due to prior extensive abdominal surgery, refractory peritonitis, or severe obesity during the initial years of the program. Percutaneous placement by IN versus IR was based on patient insurance and provider availability. This retrospective study was reviewed and approved under exempt status by the University of Texas Health at San Antonio Institutional Review Board (IRB No. 22-431E). A waiver of informed consent was granted in accordance with institutional policies and applicable federal regulations. The study was conducted in accordance with the Declaration of Helsinki. Additionally, it is confirmed that our PD program adhered to contemporary International Society for Peritoneal Dialysis (ISPD) guidelines for peritonitis prevention, catheter care, patient training, and treatment protocols throughout the study period.

A total of 93 patients were initiated on PD during the study period. Three patients were excluded because of lack of access to medical records or information about the operator. Twenty-one of the 90 patients had catheters placed twice, and six patients required a third catheter over the course of their ESKD, yielding data for a total of 117 catheters.

### Description of insertion technique, catheter type and initiation procedures

All procedures for percutaneous catheter placement were performed in an angiography suite under sterile conditions using full barrier precautions and chlorhexidine skin antisepsis. Moderate conscious sedation (midazolam and fentanyl) was administered with continuous hemodynamic monitoring. Initial ultrasound imaging was used to identify a safe peritoneal access window. Peritoneal access was obtained under real-time ultrasound guidance using a needle, followed by guidewire insertion and serial tract dilation, consistent with the Seldinger technique. A subcutaneous tunnel was then created, and the catheter was advanced using a tunneling device. A peel-away sheath was subsequently used to facilitate catheter insertion into the peritoneal cavity. Final catheter position was confirmed with fluoroscopy. A purse-string suture at the deep cuff was not routinely utilized in our technique. The catheter was secured externally with nonabsorbable sutures and an adhesive anchoring device, and the skin entry site was closed with tissue adhesive. Both IR and IN operators followed this standardized protocol at our institution, with no significant variation in technique between operator groups.

At our institution, all PD catheters were swan-neck, double-cuff Tenckhoff catheters. Information about straight versus coiled intraperitoneal segments was not consistently documented in the procedural records and abdominal imaging was not routinely available for all patients, limiting our ability to reliably determine catheter tip configuration retrospectively.

At our center, percutaneous catheters were utilized in both unplanned (urgent-start) and planned-start settings, whereas surgically placed catheters were almost exclusively performed in a planned context. In routine practice, PD training was typically initiated two weeks following catheter placement, including in unplanned-start cases when clinically feasible. However, in selected patients with uremia requiring urgent initiation of dialysis, PD training had been initiated earlier than two weeks with an accelerated training schedule; thus, meeting the definition of urgent start; however, due to logistical constraints, we did not routinely implement classical urgent start using low-volume supine PD within the center itself.

### Data collection and outcomes

We collected baseline characteristics of the study population at the time of PD initiation (age, sex, BMI, race, cause of ESKD, and comorbidities). Additionally, charts were reviewed for catheter-related problems over the course of the catheter’s life, survival of the catheter, and reasons for catheter removal. To obtain complete data, an extensive review of the individual patient records, including narratives from each monthly comprehensive visit, interdisciplinary team meeting, nursing notes, body fluid analysis and microbiology data, imaging, and procedures, was conducted.

The primary outcome was individual catheter survival, defined as the time from the start of PD training to PD termination, based on charted discontinuation of therapy with the catheter. When the exact date of cessation was unavailable, the date of catheter removal was used as a proxy. The causes of PD termination were recorded, including mechanical (catheter dysfunction, migration, adhesions, or dialysate leaks), PD-related infection (recurrent or refractory peritonitis and exit-site infection), transplant, death, patient-related psychosocial factors (burnout, loss of family assistance, non-adherence, transfer to another city or facility, and transfer to long-term rehabilitation with no PD capabilities), and PD-related factors (inadequate dialysis, hydrothorax, or recovery of kidney function).

The secondary outcome was catheter-related adverse events that did not result in catheter removal. These were divided into categories of infectious and mechanical events. Mechanical complications were defined as episodes of self-limited peri-catheter leaks, bleeding, slow drainage, or migration. Each complication episode was confirmed by a second reviewer and adjudicated by a faculty member.

To calculate the peritonitis rates per group, we first summed the total days of therapy the patients included in this analysis were on PD over the follow-up period and then converted those days into years. We then summed the episodes of peritonitis and divided the total number of episodes by the duration of therapy to calculate the rate per patient-year for the percutaneous and surgical groups separately.

### Statistical analysis

For analysis purposes, the catheters were divided into two cohorts: percutaneous and surgical groups. Patient descriptive statistics are presented. Continuous variables are presented as mean ± standard deviation or median (interquartile range), as appropriate, and categorical variables as frequencies and percentages. Distribution normality was assessed using the Shapiro–Wilk test, and non-normally distributed variables were additionally subjected to Box–Cox transformation for secondary analysis. Catheter survival was described using the Kaplan–Meier method. Catheters that remained functional at last follow-up were treated as censored observations. Given the non-randomized nature of catheter selection and the potential for confounding by indication, analyses were considered descriptive, and no formal statistical comparisons between groups were emphasized. Median survival times with 95% confidence intervals were estimated from the Kaplan–Meier curves to characterize the precision of survival estimates. Additionally, a sensitivity analysis was performed restricting the cohort to first catheter placements to account for potential non-independence arising from repeat catheter placements within the same patient. The data analysis for this study was generated using Real Statistics Resource Pack software (Release 8.8.1) and copyright (2013–2023) Charles Zaiontz.

## Results

### Patient characteristics

During the study period, total number of active prevalent patients at the end of each study year was 30, 31, 34, 37, 34, 32, 37, 34, 30, respectively. This corresponded to an average census of 33.2 patients per month. Patients in our program were routinely initiated on continuous ambulatory PD (CAPD) for approximately the first four weeks to facilitate familiarity and training with CAPD. Following this initial period, patients were offered transition to automated PD (APD), and all patients in this cohort elected to transition and subsequently remained on APD therapy. Table 1 shows the baseline characteristics of 90 patients. Of these, 25 patients had an initial catheter placed surgically, and 65 percutaneously (IR = 46 and IN = 19). A history of hernia (*n* = 4), prior abdominal surgery (*n* = 11), and obesity (*n* = 6) were the predominant reasons for the surgically placed catheter. Baseline demographic characteristics were generally similar between groups; however, patients undergoing surgical catheter placement had higher prevalence of obesity, prior hernia, prior abdominal surgery, and prior refractory peritonitis, reflecting clinical selection factors that influenced catheter placement approach in this non-randomized cohort.

**Table 1.** Baseline characteristics of study population.

		Total (*n* = 90)	Percutaneous (*n* = 65)	Surgical (*n* = 25)
Demographics	Age (years)	46.5 ± 13.7	46.9 ± 13.6	45.4 ± 14
	Female, *n* (%)	34 (37.8)	24 (36.9)	10 (40.0)
	BMI (kg/m^2^)	30 ± 6.8	29.3 ± 6.1	31.7 ± 8.1
	Hispanic, *n* (%)	68 (75.6)	51 (78.5)	17 (68.0)
	Non-Hispanic White, *n* (%)	14 (15.7)	7 (10.8)	7 (28.0)
	Non-Hispanic Black, *n* (%)	3 (3.3)	3 (4.6)	0 (0)
	Asian, non-Hispanic, *n* (%)	5 (5.6)	4 (6.2)	1 (4.0)
Cause of ESKD	Diabetes mellitus, *n* (%)	57 (63.3)	44 (67.7)	13 (52.0)
	Hypertension, *n* (%)	9 (10.0)	5 (7.7)	4 (16.0)
	Failed transplant, *n* (%)	4 (4.4)	3 (4.6)	1 (4.0)
	Congenital kidney disease, *n* (%)	4 (4.4)	0 (0)	4 (16.0)
	Glomerular disease, *n* (%)	16 (17.8)	14 (21.5)	2 (8.0)
Comorbidities	Diabetes mellitus, *n* (%)	61 (67.8)	47 (72.3)	14 (56.0)
	Hypertension, *n* (%)	87 (96.7)	64 (98.5)	23 (92.0)
	Obesity, *n* (%)	45 (50.0)	28 (43.0)	17 (68.0)
	Congestive heart failure, *n* (%)	10 (11.1)	7 (10.8)	3 (12.0)
	Previous hernia, *n* (%)	11 (12.2)	4 (4.8)	7 (21.9)
	Previous abdominal surgery, *n* (%)	36 (40.0)	22 (33.8)	14 (56.0)
	Previous refractory peritonitis, *n* (%)	15 (16.7)	9 (13.8)	6 (24.0)

**Figure 1.**
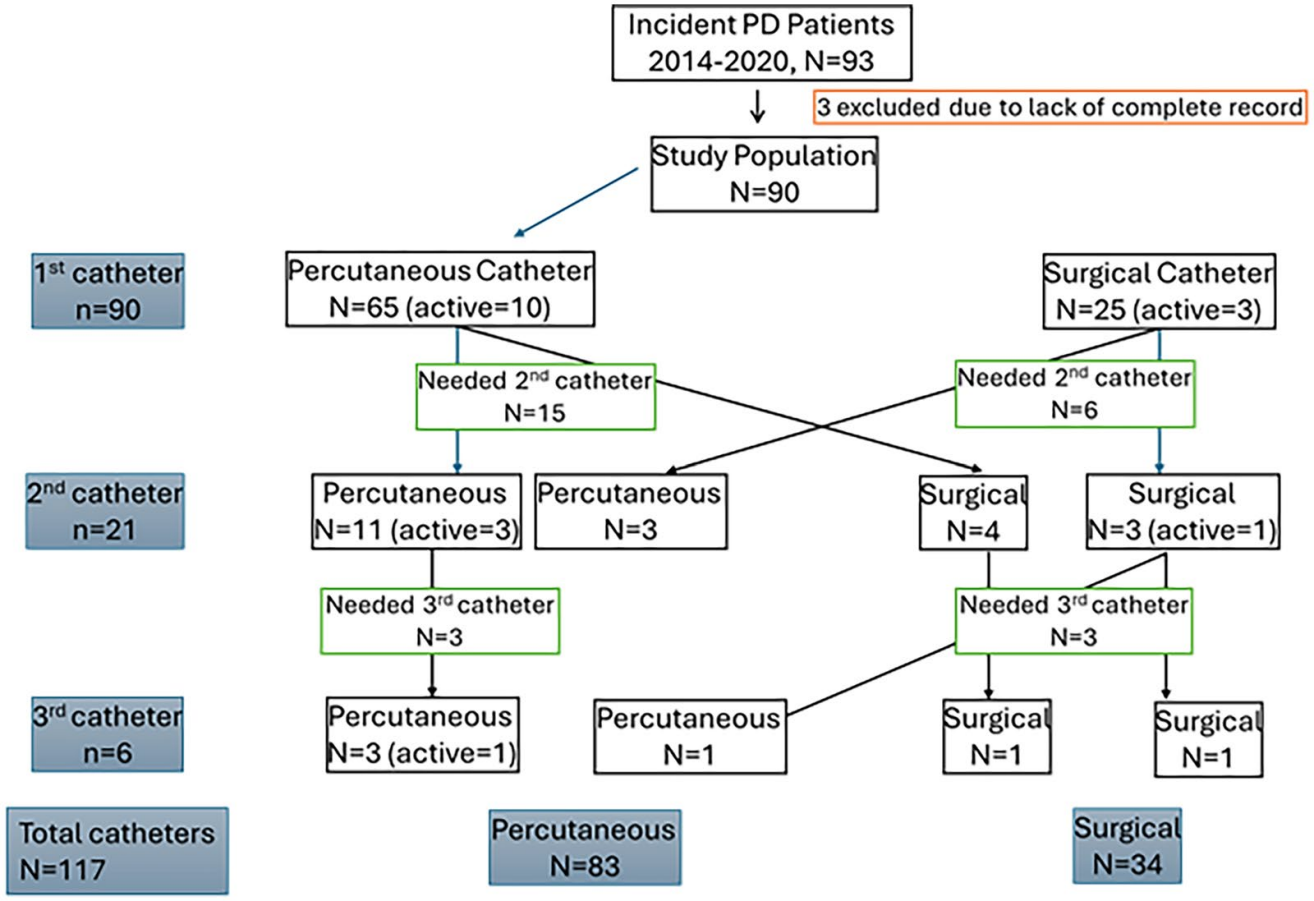
The diagram illustrates initial catheter placement approach (percutaneous versus surgical), subsequent need for second and third catheter placements, and distribution of catheter placement modality across repeat procedures. Active catheters at the end of follow-up are indicated within each subgroup.

Twenty-one of the 90 patients required a second catheter (15 in the percutaneous and six in the surgical group). The cause of catheter removal in these 21 patients was PD-related refractory infection in 12 and catheter-related issues in 9. Six patients required a third catheter (three in the percutaneous group and three in the surgical group). The cause of catheter removal was infection in three and catheter dysfunction in three patients. Thus, we recorded a total of 117 catheters placed in 90 patients: percutaneous placement (*n* = 83; 52 by IR and 31 by IN) and surgical placement (*n* = 34) (Figure 1).

### Primary outcome

**Figure 2.**
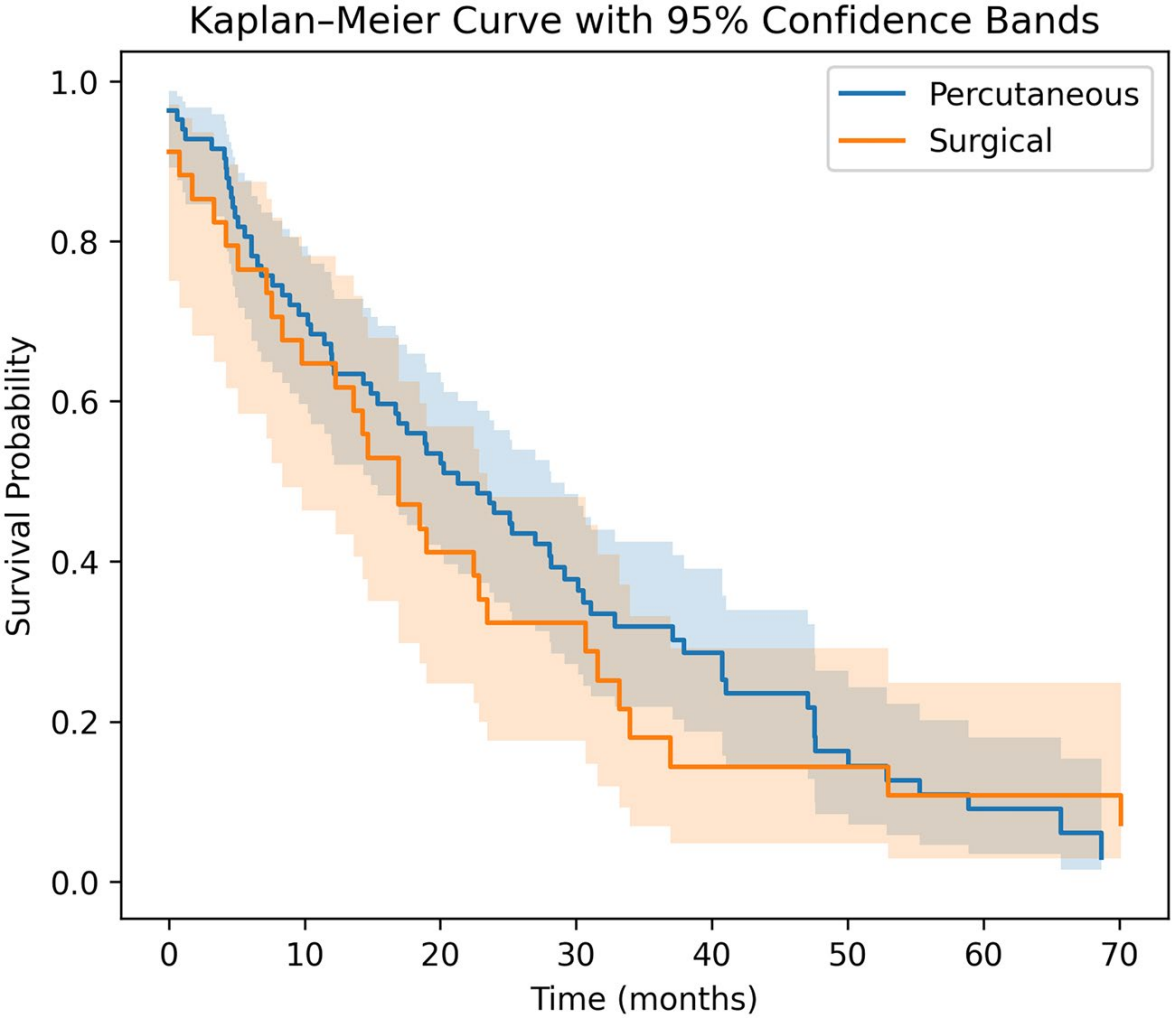
Kaplan–Meier curves demonstrating catheter survival for percutaneous and surgically placed catheters. Shaded areas represent 95% confidence intervals. Catheters that remained functional at last follow-up were treated as censored observations. The number of patients at risk decreases over time, resulting in wider confidence intervals at later time points.

**Figure 3.**
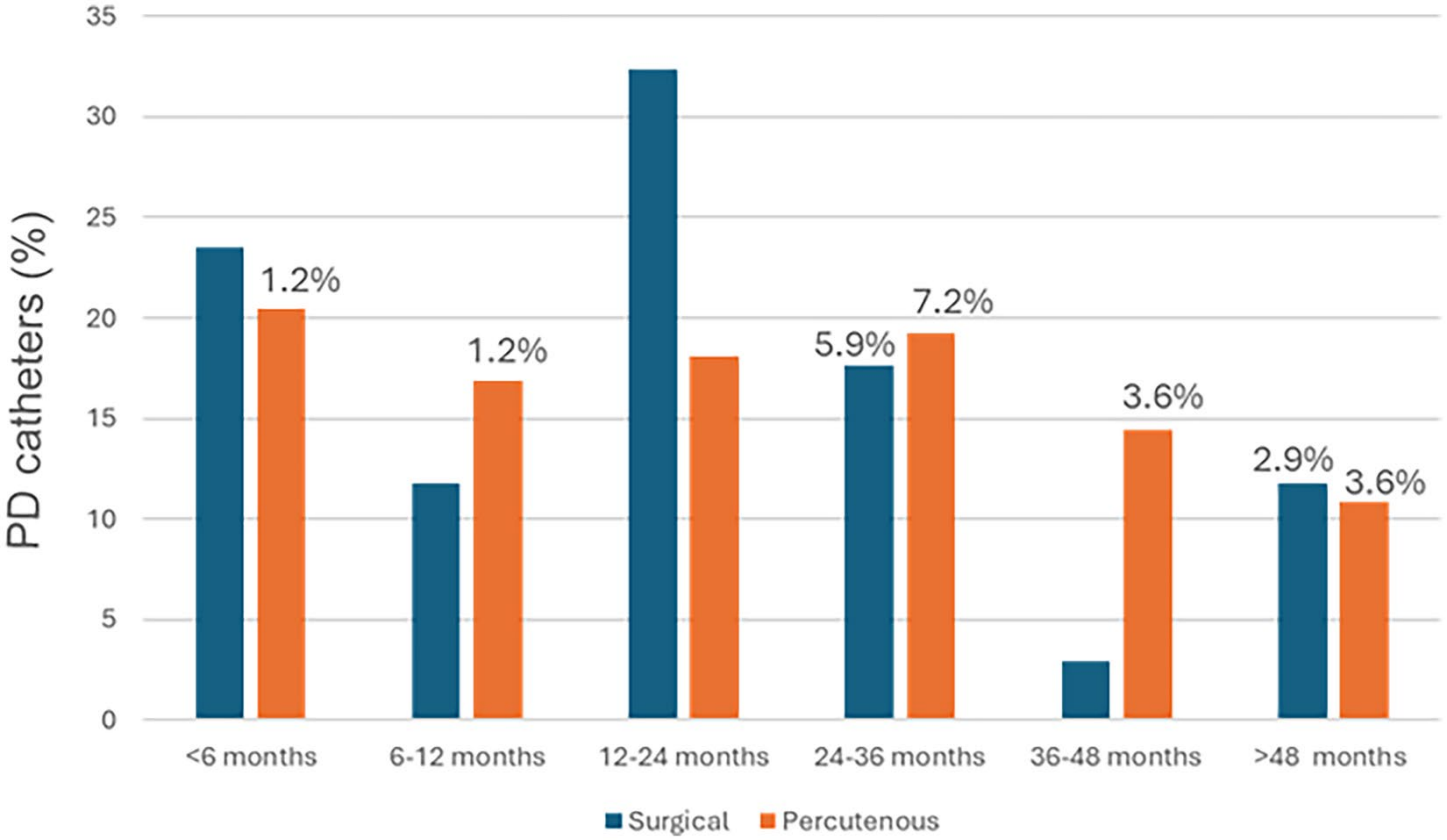
Distribution of PD catheter survival duration by catheter placement approach. The figure demonstrates the proportion of surgically and percutaneously placed PD catheters remaining functional across predefined catheter survival intervals. Percutaneously placed catheters demonstrated a broader distribution of long-term catheter survival comparable to surgically placed catheters.

By the end of the study follow-up period, 14 (16.9%) catheters in the percutaneous group and three (8.8%) catheters in the surgical group were actively used to pursue PD therapy. Three catheters in each group could not be ever used and removed, and the causes are outlined in the next paragraph. Median survival of individual catheter determined from PD start to cessation or end of study follow-up was 20.3 (IQR; 6.8, 36.0) months for the percutaneous and 17.0 (IQR; 7.2, 30.8) months for the surgical group. A sensitivity analysis restricted to first catheter placements, the median catheter survival remained comparable at 20.0 (IQR 6.8–32.9) months for percutaneous placement (*n* = 65) and 14.3 (IQR 4.7–29.6) months for surgical placement (*n* = 25), consistent with the findings of the primary analysis. Figure 2 represents Kaplan–Meier curves demonstrating catheter survival for percutaneous and surgically placed catheters. Figure 3 demonstrates the proportion of total catheters used for durations of <6 months, 6–12 months, 12–24 months, 24–36 months, 36–48 months, and >48 months in bar graph.

### Reasons for catheter removal

The reasons for the catheter removal are listed in Table 2. PD-related infections, such as recurrent or refractory peritonitis and exit-site infection, were the leading causes of catheter removal in the percutaneous (21.7%) and surgical (29.4%) groups. The second most common cause of PD termination was patient-related psychosocial factors in the percutaneous group (22.9%) and mechanical reasons and transplant in the surgical group (17.5%). One catheter in each group was never used and was removed due to a change in decision for dialysis modality after catheter placement. Additionally, two catheters were removed soon after placement in the percutaneous group, one due to pericatheter leak with fluid pocket and and other due to catheter malfunction secondary to omental wrapping. The surgical group also had two nonfunctional catheters, one due to adhesions and the other due to wrong tunnel direction causing migration of the catheter in the hepatic flexure. None of the deaths in either group were related to direct complications of PD (cardiac, *n* = 7; mesenteric ischemia, *n* = 1; pulmonary hypertension, *n* = 1, COVID = 2; failure to thrive with lupus complications, *n* = 1; end-stage liver failure, *n* = 1; and malignancy, *n* = 1).

**Table 2.** Reasons for catheter removal.

Causes, *n* (%)	Total (*n* = 117)	Percutaneous (*n* = 83)	Surgical (*n* = 34)
Mechanical	18 (15.4%)	12 (14.5%)	6 (17.5%)
-Catheter malfunction	13 (11.1%)	9 (10.8%)	4 (11.8%)
-Abdominal wall defect	5 (4.3%)	3 (3.6%)	2 (5.9%)
PD-related infection	28 (23.9%)	18 (21.7%)	10 (29.4%)
Transplant	12 (10.3%)	6 (7.2%)	6 (17.6%)
Death	14 (12.0%)	9 (10.8%)	5 (14.7%)
Patient-related factors	23 (19.7%)	19 (22.9%)	4 (11.8%)
PD-related factors	5 (4.27%)	5 (6.0%)	0 (0%)
*Active*	*17 (14.5%)*	*14 (16.9%)*	*3 (8.8%)*

These italic catheters were active and still in use by the end of the study period.

### Peritonitis and infectious complications

PD-related peritonitis occurred in 29 of the 83 (34.9%) percutaneous catheters. Four catheters had two episodes and six catheters three episodes, yielding a total of 45 episodes in 83 catheters (54.2%). Peritonitis occurred in 17 of 34 (50%) surgically placed catheters. Three catheters sustained two episodes, and one catheter sustained three episodes, yielding a total of 22 episodes in 34 catheters (64.7%). Taking account the time patients in each group were in treatment, overall peritonitis rate was 0.295 episodes per patient-year or 1 episode per 40.7 patient-months. The peritonitis rate was 0.275 episodes per patient-year in the percutaneous group (approximately one episode per 43.6 patient-months) and 0.345 episodes per patient-year in the surgical group (approximately one episode per 34.8 patient-months). Peritonitis microbiology was broad and overall similar between the surgical and percutaneous catheter groups. Episodes of culture-negative peritonitis occurred 1 in the surgical group and 6 in the percutaneous group. The most frequently isolated organisms were *Pseudomonas aeruginosa* (3 vs. 10 episodes), coagulase-negative *Staphylococcus* (2 vs. 9 episodes), and *Klebsiella* species (2 vs. 3 episodes) in the surgical and percutaneous groups, respectively. *Staphylococcus aureus* occurred in 2 surgical and 1 percutaneous episode, while diphtheroid was identified in 2 episodes in each group. Streptococcal species accounted for 1 and 3 episodes, respectively. Acinetobacter-related peritonitis occurred only in the surgical group (*n* = 2), whereas *Escherichia coli* and *Serratia* species occurred only in the percutaneous group (*n* = 1 each). *Enterobacter* species occurred in 1 episode in each group. *Leclercia adecarboxylata*, *Pantoea* species, and *Pasteurella multocida* were each identified in 1 surgical patient. Rare organisms identified exclusively in the percutaneous group included *Micrococcus*, *Citrobacter*, *Proteus*, *Candida*, and *Rothia* species, with one episode each. Polymicrobial peritonitis occurred in 1 surgical and 3 percutaneous episodes. Two additional episodes in the surgical group involved unidentified species.

### Other mechanical complications

In addition to major mechanical complications requiring PD catheter removal, as mentioned above, other minor mechanical complications were noted. Slow drain occurred on several occasions in 16 catheters in the percutaneous group (23.9%) and five catheters in the surgical group (14.7%). These were mostly related to constipation and improved with proper use of laxatives. Three catheters in the percutaneous group experienced migration, two needed surgical interventions, and one improved with laxatives. In the surgical group, one catheter migrated high into the pelvis and improved with laxatives. Catheter malfunction due to omental wrapping occurred with three catheters in the percutaneous group and required surgical intervention without needing a new catheter placement. The corresponding number in the surgical group was two, and both improved with surgical intervention. Other than one early pericatheter leak in the percutaneous group, none of the catheters in either group experienced this complication. The proportions of each type of mechanical complication were similar between the two groups.

## Discussion

In this single-center observational study, we report long-term outcomes of percutaneously placed PD catheters in a contemporary PD program. To our knowledge, this represents one of the most extensive modern evaluations of percutaneous catheter durability with extended follow-up. The median catheter survival was 20.3 months (IQR 6.8–36.0) for percutaneously placed catheters and 17.0 months (IQR 7.2–30.8) for surgically placed catheters with 14.5% of catheters remaining in use at the end of study period. A sensitivity analysis restricted to first catheter placements yielded similar findings. The non-randomization design of the study precludes a comparison analysis between the groups. However, this duration is very similar to that of PD treatment reported in the PD population with predominant use of surgically placed catheters. Moreover, we observed similar rates of PD-related infections and mechanical complications compared to the surgical group and what has been reported in the literature. Overall, we show that percutaneous catheters can be safe and effective not only in the short term, but also in the long term.

Traditionally, well-established high-volume PD programs rely heavily on surgeons, and long-term catheter outcome data are mostly based on surgically placed catheters. Over the last two decades, percutaneously placed catheters have increasingly been utilized; however, in the largest dataset including Medicare insured adults >18 years undergoing PD catheter insertion, the proportion of PD catheters placed percutaneously increased from 5% in 2007 to only 6.5% (by IR = 3.6% and by IN = 2.9%) for time period between 2010 and 2019 [9,10]. Several recent studies have compared the influence of catheter placement methods on catheter survival and complications and have been supportive of percutaneous placement of PD catheters; however, all these studies reported outcomes at 3–12 months post-insertion [11–13]. A contemporary UK study comparing 325 patients with percutaneously inserted catheters versus 444 patients with surgically inserted PD catheters found no significant difference in catheter-related events, infection, or removal at 1 year follow up between the two methods of PD catheter insertion [7]. An unproven concern about long-term success remains one of the major causes of underutilization of these catheters because the evidence for long-term outcomes is sparse [14]. Our experience helps fill this knowledge gap by demonstrating the viability of percutaneously placed catheters for sustained PD therapy over a longer time period.

The most recent International Society for PD guidelines for creating and maintaining optimal PD access in adult patients state that the PD catheter implantation approach should be ‘based on patient factors, facility resources, and operator expertise’ [6], supporting a complementary approach using both surgically and percutaneously placed catheters. The minimally invasive nature of percutaneous placement offers benefits such as shorter procedure time, avoidance of general anesthesia, reduced intensive operative resources, faster post-procedure recovery, and remains an attractive option for frail, comorbid patients, particularly those in need of urgent PD catheter placement [15]. The advantages of the surgical laparoscopic technique are the direct visualization of the abdomen and the ability to perform additional, advanced procedures such as lysis of adhesions and omentopexy with the intent of minimizing PD catheter flow dysfunction and complications [9]. This may be particularly advantageous in certain subsets of patients, such as those with adhesions from prior surgery or an existing hernia. At our center, we serve a population that is predominantly present in the emergency room in the terminal stages of CKD without pre-dialysis care. The availability of an interested interventional program enabled prompt catheter placement for patients opting for PD, many of whom would otherwise have defaulted to in-center hemodialysis with tunneled central venous catheters. At the same time, we could maintain surgical collaboration for selected patients requiring advanced intra-abdominal interventions. This complementary approach contributed the rapid growth and maintenance of our program which had been previously reported [16]. Similarly, another program reported 117% growth when catheter insertion was performed by nephrologists [17].

Peri-catheter leakage, migration, and catheter malfunction are major concerns for percutaneously placed catheters. However, advancements and innovations in technology have increased the durability by addressing these concerns [15]. For example, the minimally invasive IR technique, with a small PD catheter insertion site through the parietal peritoneum, combined with the ability to place a purse-string suture around the deep cuff and pericatheter tissues within the rectus abdominus muscle, decreases the risk of dialysate leakage and allows urgent-PD. Image-guided placement of the catheter in the retrovesical or retro-uterine space prevents migration of the catheter [15]. In our study, there was no significant increase in mechanical complications in the percutaneously placed catheter group. The causes of catheter removal were similar between the groups, with infection being the most common and psychosocial factors the second most frequent. These findings suggest that, insertion technique had little to no meaningful impact on long-term catheter survival. Additionally, the percutaneous group had a lower rate of peritonitis than the surgical group, but in the setting that patients with refractory peritonitis had their repeat catheter placed surgically, there was an indication bias. This observation is consistent with the findings of previous studies, which suggested a potential advantage of percutaneous placement in reducing peritonitis rates, as the minimally invasive approach may be associated with reduced infection risk [8,10]. Another striking finding was the success of percutaneous PD catheter placement in patients with obesity. Although the surgical group had a higher proportion of patients with obesity (68%) and higher BMI, the percutaneous group also had 43% of patients with obesity, similar to the prevalence of obesity in the US population, highlighting that the percutaneous technique can meet the needs of the population with increasing rates of obesity. Of note, a study by Xie et al. [18] also illustrated that, irrespective of BMI, percutaneous catheter placement was a viable option when compared to surgery.

Despite the valuable insights provided by this study, it is essential to acknowledge its limitations. The major limitation is that this was a single-center cohort study with a retrospective and non-randomized design. However, our real-world results demonstrate the durable longevity of percutaneously placed PD catheters, supporting their viability as a minimally invasive option to provide optimal PD access and outcomes. Second, we present the survival of the individual catheter and not the patient on a PD. We had to follow this approach, as one patient could have a repeat catheter placed surgically or vice versa. The median duration of individual percutaneous catheter placement was 20 months, which is comparable to 27.6 and 18 months reported previously in two studies for median time on PD [19,20]. These reports included both surgically and percutaneously placed catheters as well as multiple catheters in the same patients while assessing the duration of PD. Third, due to a transition in the electronic medical record system during the study period, a reliable assessment of break-in intervals and urgent-start PD utilization was limited by incomplete and inconsistent documentation of catheter insertion and PD initiation dates. Fourth, detailed characterization of intraperitoneal catheter tip configuration (straight versus coiled) was not consistently available in procedural records or confirmatory imaging, limiting assessment of potential catheter design-related effects on outcomes; however, prior studies have demonstrated inconsistent differences in the outcome between these configurations, and all patients in our cohort received uniform swan-neck, double-cuff Tenckhoff catheters, minimizing the impact of catheter design variability on outcome. Fifth, we were unable to reliably report exit-site and tunnel infection rates separately because these data were not consistently or systematically documented in the retrospective medical records throughout the study period. Lastly, our study population was derived from a predominantly Hispanic, safety-net cohort with a high prevalence of diabetes and comorbid conditions, which may limit generalizability to other PD programs with different patient demographics. However, the favorable long-term catheter outcomes observed in this higher-risk population suggest that percutaneous PD catheter placement can be successfully implemented in clinically complex settings. These findings may support the broader applicability of this approach across diverse practice environments. Nonetheless, further studies in varied populations are warranted to confirm external validity.

In conclusion, we have demonstrated that percutaneous placement of PD catheters is a viable alternative for long-term PD therapy. Our experience emphasizes that the choice of insertion technique for catheter placement should be guided by patient-specific factors, as well as local expertise, and resources. Larger multicenter studies are needed to confirm these findings, which have implications for PD programs to allocate resources appropriately. Careful consideration of the optimal division of labor and coordination between specialties placing PD catheters can be a way to meet the growing demand for PD and improve the uptake of PD modalities.

## Data Availability

The data that support the findings of this retrospective study are available from the corresponding author upon reasonable request due to privacy restrictions. The data were not publicly available to protect the confidentiality of study participants.
